# Application interface design of Chongqing intangible cultural heritage based on deep learning

**DOI:** 10.1016/j.heliyon.2023.e22242

**Published:** 2023-11-11

**Authors:** Yanlong Liu, Peiyun Cheng, Jie Li

**Affiliations:** a School of Art, Chongqing Technology and Business University, Chongqing 400067, China; b Art of College, Kunsan Nation University, Kunsan 540541, South Korea; c Tourism Management Department, PaiChai University, Daejeon 340934, South Korea

**Keywords:** Urbanization, Smart city, Intangible cultural heritage, Application, Deep learning

## Abstract

In order to integrate the concept of intangible cultural heritage (ICH) protection into the construction of smart cities, realize the organic integration of smart cities and cultural heritage, and improve the cultural experience of urban residents and tourists, this study explores an interactive design scheme of smart cities application interface applied to ICH protection to meet the needs of protection and inheritance. Firstly, the ICH of Chongqing is sorted out and classified. The ICH-related APP interfaces in the market are analyzed through investigation. Secondly, an image recognition algorithm of ICH based on deep learning (DL) technology is proposed and applied in APP to realize automatic recognition and introduction of ICH. Finally, a set of APP interface interaction design schemes is designed based on user habits and visual feelings to enhance user experience. The experimental results reveal: (1) The model for recognizing ICH images using the convolutional neural network (CNN) has higher recognition accuracy, recall, and F1 value than the model without CNNs; (2) After incorporating transfer learning (TL) into the model, the recognition accuracy, recall, and F1 value of the model have further improved; (3) The survey results show that the Chongqing ICH APP interface system based on DL technology, user habits, and visual perception performs better in terms of user experience, usability, and other aspects. This study aims to design an APP interface system for the Chongqing ICH based on DL technology, user habits, and visual perception, to improve user experience and usability. Future research directions can further optimize image recognition algorithms to improve ICH's recognition accuracy and efficiency. Meanwhile, new technologies, such as virtual reality, are combined to enhance users' interactive experience and immersion.

## 1 Introduction

Intangible cultural heritage (ICH) has increasingly gained universal recognition in recent years as a crucial component of inheriting historical culture and cementing social identity [1]. However, these precious ICH are often presented in the form of images, and how to efficiently and accurately identify and protect them has become a challenging issue [2]. Meanwhile, with the popularity of smartphones and mobile internet, application (APP), as a convenient way of cultural dissemination and experience, is gradually changing how people access information and culture. In this context, it is practical significance to develop an APP interface system of ICH based on deep learning (DL) technology to enhance user experience and usability [3].

However, the recognition of ICH images faces multiple complex scenarios, such as lighting changes, scale transformations, and object occlusion, which puts forward higher requirements for image recognition technology [4]. Meanwhile, the limited size of mobile device screens and the need for user experience also pose challenges to the design of app interfaces [5]. Therefore, this study aims to apply DL technology to improve the accuracy and efficiency of ICH image recognition through methods such as the convolutional neural network (CNN) and transfer learning (TL). Moreover, this study combines DL technology with user habits and visual perception to design an APP interface system of ICH that can enhance user experience and usability. The motivation of this study is to fully realize the importance of ICH and the potential of digital technology for its dissemination and protection. By adopting DL technology, this study builds a new way for users to interact with ICH, thus stimulating more people's interest in these precious cultures and passing them on to future generations.

In this context, this study is innovative. Firstly, the ICH's image recognition model based on CNN and TL is developed to improve the accuracy, recall, and F1 value of image recognition of ICH. Secondly, the Chongqing ICH APP interface system is designed based on user habits, DL technology, and visual feelings to advance user experience and usability. Finally, the application of a questionnaire on users' influence on APP interface design is explored, thus providing a reference for the further optimization of APP design. This comprehensive research method is of great significance for improving the effectiveness of ICH protection and inheritance.

The research structure of this study is as follows. Firstly, section 2 analyzes and summarizes domestic and international relevant research; Secondly, section 3 introduces relevant theories, including DL technology, CNN, etc., and the design and implementation of an ICH image recognition model based on DL technology are elaborated in detail; Section 4 presents experimental results and data analysis to verify the effectiveness of the proposed method. Finally, Section 5 summarizes the entire study and looks forward to future research directions. It is believed that this study may bring new ideas and approaches for the prevention and transmission of ICH.

## 2 Literature review

With the advancement and popularization of DL technology in recent years, the discussion on the protection and inheritance of ICH based on DL has steadily drawn the attention of the academic community and society [6].

### 2.1 ICH development with AI

AI research in the subject of ICH protection has also made significant progress. Hu (2023) applied image recognition technology to protect and inherit traditional ICH crafts. Constructing a DL model tailored to specific processes makes it possible to automatically identify and describe complex process steps, promoting the inheritance and innovation of traditional process techniques [7].

In addition, the digital preservation and virtual inheritance of cultural heritage have also become a hot research topic in China. Katifori et al. (2023) utilized 3D scanning technology and virtual reality technology to digitize physical cultural heritage into virtual models, enabling them to be preserved and displayed. This digital approach not only preserves the original appearance of cultural heritage, but also provides viewers with an immersive experience, promoting the dissemination and popularization of ICH [8].

### 2.2 CNN

The first was the image recognition investigation of ICH by DL technology based on this [9]. Aiming at the image recognition of ICH, scholars proposed many DL-based methods, such as CNN-based image classification methods, Recurrent Neural Network (RNN)-based sequence classification methods, etc. [10,11]. These methods achieved certain research findings in the ICH image recognition of and improved the efficiency and accuracy of its protection and inheritance [12]. The second was a study of ICH digitization using the DL technology [13,14]. Scholars proposed DL-based ICH digitization methods, such as DL-based audio and video processing [15,16]. These methods can better preserve and inherit ICH and improve its efficiency [17]. Additionally, the DL technology-based protection and inheritance research of ICH also included the construction of a knowledge graph and intelligent recommendation of ICH [18,19]. These research directions had broad application prospects and significance in the protection and inheritance of ICH [20]. Azmi et al. (2023) used neural network to automatically identify the notes of traditional music, realized sound classification through CNN, and generated the melody of traditional music by RNN. Their research showed that neural networks were widely used in protecting and promoting ICH [21]. Lee (2022) paid attention to the application of neural network in protecting indigenous languages. By using LSTM networks to automatically translate indigenous language texts and using CNN for speech recognition, oral traditions can be preserved [22]. Tao et al. (2022) discussed the potential role of neural network in the digitization and analysis of traditional dance forms. They used the improved back propagation neural network to automatically identify different dance movements and dance performances, train and optimize the model, and combine video data with cultural background to realize the protection and promotion of ICH [23].

### 2.3 TL

The research and application of TL in the ICH protection field gradually attracted international researchers' attention. The researchers applied TL to image recognition and classification tasks to improve the recognition accuracy of ICH elements and the model's generalization ability. For example, Ata and Başar (2023) can achieve good results with a small number of samples by migrating pre-trained models to ICH image recognition tasks, providing strong support for the automatic recognition and classification of ICH elements [24].

Furthermore, TL was also applied to feature extraction and representation learning of ICH data. For instance, Katelieva and Muhar (2022) migrated existing cultural heritage data and knowledge to the museum guide system to construct more effective feature representations, thereby improving the performance of the model under small samples [25]. By combining knowledge from several craft datasets, Ma et al. (2022) made it possible for diverse crafts to be automatically recognized and classified [26].

To sum up, although previous studies have made some progress in the protection of ICH, there are some obvious shortcomings. Firstly, previous studies usually did not fully consider the size of datasets and the diversity of samples, which may lead to insufficient data, thus affecting the accuracy and generalization ability of the model. Secondly, the diversity and cultural background of ICH were rarely involved in previous studies, and intangible elements in different regions and cultures may need different methods and personalized solutions. Finally, the consideration of ethical issues has not been paid enough attention in the previous literature, which is very important for the digitalization, protection and privacy maintenance of ICH. Under this background, this study closely combines DL technology with ICH protection, and uses CNN and TL to improve the identification and protection effect of intangible elements. Meanwhile, this study also pays attention to the digital display and user experience of intangible cultural protection, and improves the cultural experience and convenience of users through the APP interface system designed based on DL technology and user habits and visual feelings. By making up the blank of previous research, this study aims to provide new ideas and methods for the technical application and research in the field of ICH, thus highlighting its innovation and differences in existing research.

## 3 Research model

This section mainly focuses on the study of the image recognition model of ICH based on DL, including theoretical elaboration and model design. The theoretical part mainly includes the theory of intangible culture, the theory of APP design and the structure of CNN, while the model design part focuses on how CNN and TL can improve the image recognition performance. In general, this section provides a modern perspective and shows how to use DL technology to enhance the image recognition of ICH, which is helpful to better protect and inherit ICH.

### 3.1 Theoretical basis

(1) Theories related to the protection and inheritance of ICH

In order to provide important guiding principles for the protection and inheritance of ICH and ensure the sustainability and vitality of cultural heritage, this section discusses the theoretical content related to the protection of ICH [27].

ICH refers to various oral traditions, performing arts, social practices, traditional crafts, and related natural and cultural landscapes [28]. These cultural elements are the product of the human creative spirit. They are the integrated embodiment of the cognition, values, production methods, forms of social organization, and cultural inheritance of specific communities [29].

The main theoretical contents of the protection and inheritance of ICH are exhibited in Table 1.

(2) Relevant theories of APP interface design

**Table 1:** The main theoretical contents of the protection and inheritance of ICH.

Level	Description
Sustainability	Environmental, economic and social factors should be taken into account to ensure that the transmission and protection of ICH do not have negative environmental and social impacts [30].
Community participation	The inheritance of ICH should be based on community participation and joint efforts, rather than solely being completed by the government or professional institutions [31].
Inheritance method	Its inheritance should be diversified and can be completed through oral tradition, practice, experience, performance, education, and other ways [32].
Readability	Its inheritance should be understood and recognized, and should not be too complex or profound, making it difficult for the public to accept and understand [33].
Innovation	The inheritance should preserve not only traditional elements but also have innovative thinking and methods to adapt to the changes and needs of the times [34].

This section introduces the related theories of APP design to provide theoretical support for the following construction of Chongqing ICH APP interface interactive system.

APP interface design refers to the visual representation of various information, functions, and interactive elements designers present in an APP [35]. The relevant theories of APP interface design are outlined in Table 2.

(3) The image recognition algorithm of ICH based on DL technology

**Table 2:** The relevant theories of APP interface design.

Design types	Definition	Design principles
User experience design	User experience stands for subjective feelings users feel when using products, including the convenience of use, clarity of information, speed, and accuracy of feedback, etc. [36,37].	A concise and clear interface
		Reasonable functional layout
		Timely and accurate feedback
		Smooth and natural operation
Information architecture design	Information architecture means the process of classifying, organizing, and structuring various information and functions in an APP [38,39].	Clear classification of information
		Navigation consistency
		Clear information hierarchy
Interaction design	It refers to the process of how designers interact with users and apps, covering layout of interface elements, design of functions, presentation of interactive effects, etc. [40,41].	Easy to learn and use
		Natural operation mode
		Timely and accurate interface feedback
		Easy-to-discover and use features
Responsive design	It means that the interface of an APP can automatically adapt to different screen sizes and device types [42,43].	Adapt to multiple screen sizes and device types
Accessibility design	It indicates how a designer can make an APP useable by all people, including users with physical and cognitive disabilities [44,45].	Easy-to-understand interface
		Simple and clear operation method
		Clear and easy-to-understand information

With the continuous development and application of information technology, digital protection and inheritance of ICH has become increasingly important. Among them, image recognition technology plays an important role in the digital preservation and inheritance of ICH. Therefore, this section focuses on the image recognition algorithm of ICH based on DL technology.

**Fig. 1:**
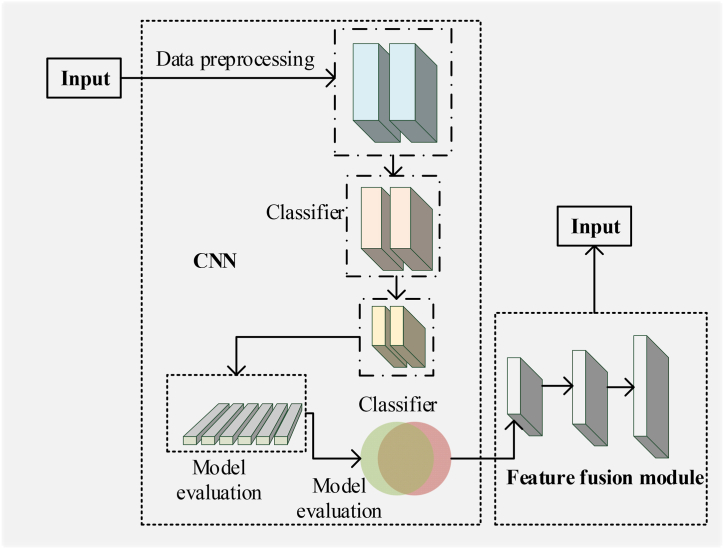
CNN structure.

The proposed algorithm principally employs CNN to extract and classify image features [46]. The structure of the CNN is revealed in Fig. 1.

Fig. 1 presents the principle and method of the CNN algorithm as follows.

1) Data preprocessing

ICH images are preprocessed, involving image size unification, gray processing, data enhancement, etc. The purpose of preprocessing is to enhance the data's diversity and robustness and improve the model's generalization ability [47].

2) Feature extraction: CNN is used for feature extraction to convert images into a multi-layer abstract feature representation. The structure of CNN encompasses convolutional, pooling, and fully connected (FC) layers. The convolutional and pooling layers can extract the local features of the image, while the FC layer can extract the global features. The operation of the convolutional layer is in equation (1):

(1) $$y_{i,j,k}=\sigma \left(\sum_{m=0}^{M-1}\sum_{n=0}^{N-1}\sum_{l=0}^{L-1}\omega_{m,n,l,k}x_{i+m,j+n,l}+b_{k}\right)$$

$y_{i,j,k}$ represents the element values of the *i*th row, *j*th column, and *k*th channel in the output feature map; $\omega_{m,n,l,k}$ indicates in the convolution kernel, the weight values of the *m*th row, *n*th column, *k*th channel, and *l*th convolution kernel; $x_{i+m,j+n,l}$ stands for the pixel values of the *i* + *m*th row, *j* + *n*th column, and the *l*th channel in the input image; $b_{k}$ means the bias value of the *k*th convolution kernel.

The operation of the pooling layer reads in equation (2):

(2) $$y_{i,j,k}=\max_{m=0}\max_{n=0}x_{i+m,j+n,k}$$

The max function represents the maximum operation.

3) Classifier design

The classifier classifies the extracted features to determine the category of the image. The classifier used in this study is softmax classifier. The equation adopted by the classifier is in equation (3) as follows:

(3) $$p_{k}=\frac{e^{zk}}{\sum_{j=1}^{K}e^{zj}}$$

$p_{k}$ and $e^{zk}$ represent the probability and score of the input eigenvector x belonging to the *k*th category; *K* refers to the total number of categories.

4) Model training

Many annotated ICH images are employed for model training, and the optimization algorithm adjusts network parameters. Thus, the network can better classify images.

5) Model evaluation

The recognition accuracy, recall, F1 value, and other model indicators are calculated to evaluate the model performance by classifying a group of test images.

6) Model optimization

The model is optimized according to the evaluation results. The model is optimized by increasing network depth, adjusting parameters, using more training data, and others, so that the model has a better effect. Furthermore, the TL algorithm is utilized to optimize the model [48,49].

(4) Integrating TL into CNN

In the process of digital protection and inheritance of ICH, DL technologies such as CNN have shown strong image recognition ability. However, in order to achieve better results in specific ICH projects, a lot of annotation data and computing resources are usually needed, which may be limited in some cases. In addition, TL algorithm can accelerate model training and improve performance by migrating weights and feature extractors from a pre-trained DL model, and at the same time, it can reduce data requirements. Therefore, in order to overcome this challenge and improve the performance of the model, this section introduces TL into CNN to improve the effect of image recognition of ICH.

The transfer of knowledge from a model that has already been trained on one task to another related task speeds up model training and improves performance [50]. In the structure of the CNN constructed above, TL is integrated into the format described in Table 3.

**Table 3:** The specific operation of integrating TL into CNN structure.

Layer	Name	Freeze weights	Fine-tune weights	New task output
Pre-training model layer	Convolutional	Yes	No	No
Pre-training model layer	Pooling	Yes	No	No
Newly added layer	Fully Connected	No	Yes	Yes
Newly added layer	Softmax	No	Yes	Yes

**Fig. 2:**
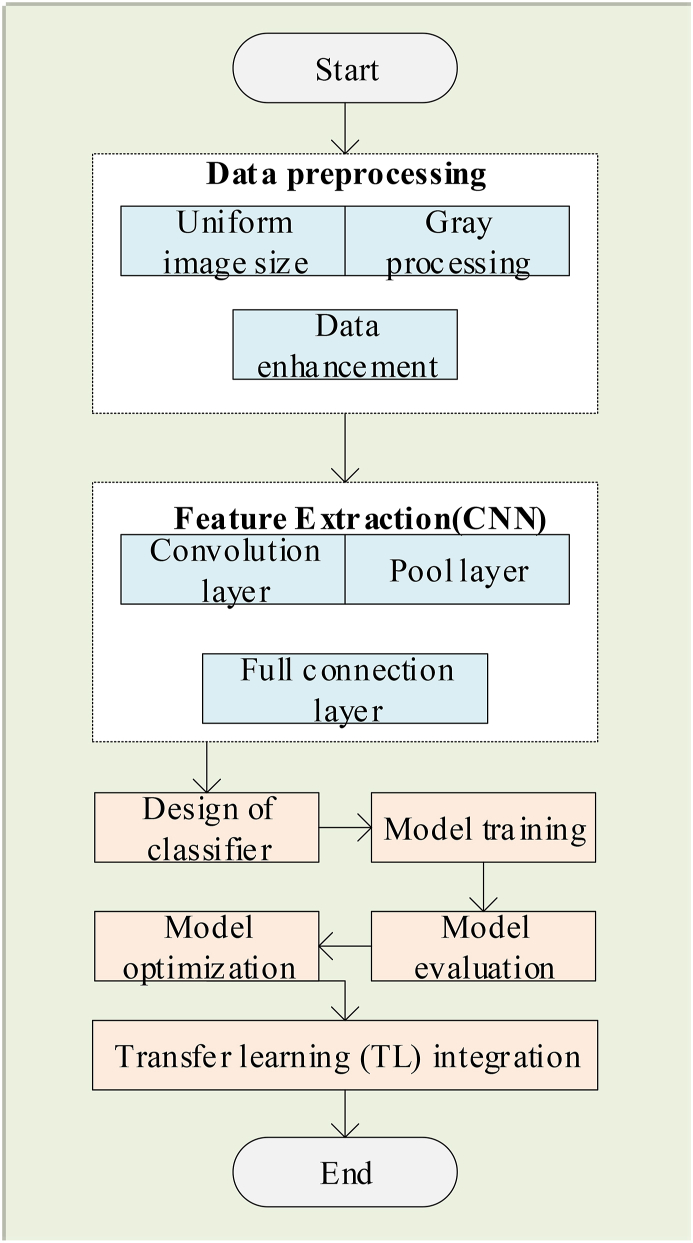
Flow chart of ICH image recognition algorithm based on DL and TL.

According to the above contents, the image recognition algorithm of ICH based on DL technology and TL constructed in this paper is shown in Fig. 2.

**Fig. 3:**
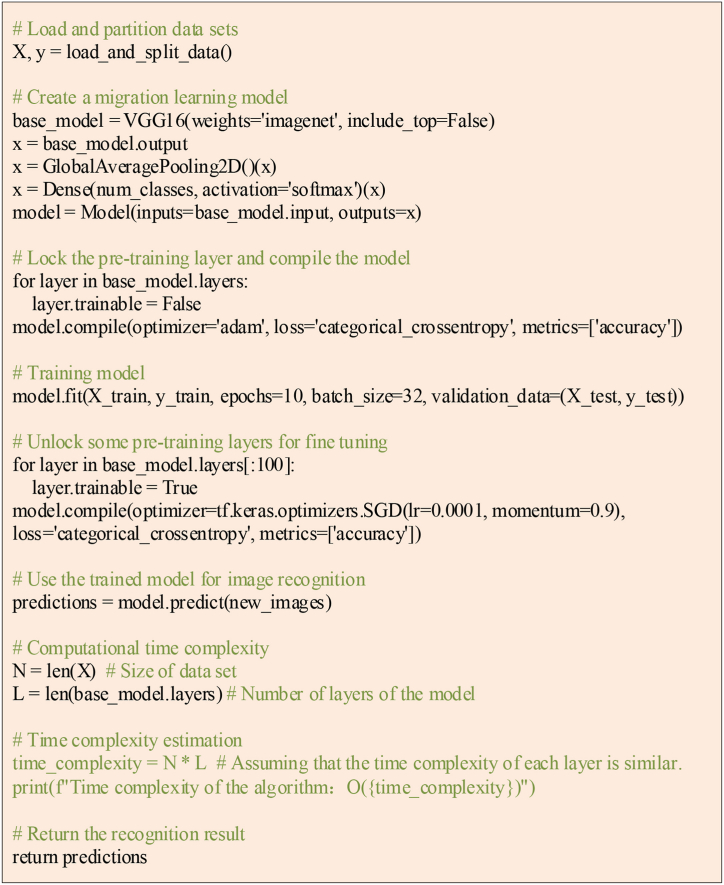
Pseudocode of ICH image recognition algorithm based on DL and TL.

In addition, Python software is used to construct and run the pseudocode of the above ICH image recognition algorithm flow based on DL and TL, and its pseudocode is shown in Fig. 3.

The pseudo-code in Fig. 3 retains the main steps of the algorithm, including loading and dividing datasets, creating a migration learning model, training and fine-tuning the model, and using the model for image recognition.

### 3.2 The chongqing ICH APP interface design scheme

(1) Research and classification of ICH

In order to better understand, protect and inherit ICH, researchers have conducted in-depth research and classification. This section focuses on the study of ICH and discusses its key role in the protection and inheritance of ICH.

The research and classification of ICH in Chongqing aim to protect, inherit, and utilize ICH resources, promote the development of the cultural industry, and improve the city's image. The principles followed by the Chongqing Cultural Heritage Protection Center during the investigation are detailed in Table 4.

**Table 4:** The principles followed by Chongqing Cultural Heritage Protection Center during the investigation.

Characteristics	Method	Principles
ICH's essential characteristics and heritage values	Focus on the deep connotation of ICH	Focus on the nature and value of ICH [51]
Be practical and realistic	A comprehensive and systematic understanding of the history, current situation, and future development trends of ICH is achieved through a combination of field investigation and in-depth research	Adhere to the truth and objective, and avoid subjective assumptions [52]
Respect and protect ICH's intellectual property rights (IPR) and national characteristics	Emphasis is placed on communication and cooperation with folk cultural inheritors and relevant institutions	Respect the rights and interests of inheritors and institutions, protect IPR and ethnic characteristics [53]
Scientific classification and refinement of ICH types and ranges	Basic data and guidance are provided for protection and utilization	A scientific and systematic classification and refinement system is established to provide basic data and guidance for protection and utilization [54]

According to the above principles, the main contents involved in the ICH survey and classification in Chongqing are illustrated in Table 5.

(2) Analysis of ICH's APP interface design in the market

**Table 5:** The main contents of the ICH survey and classification in Chongqing.

Content	Description
Summary	The definition, historical background, protection status, and challenges faced by Chongqing ICH are summarized.
Type	Classification is done according to types and characteristics, including oral tradition, performing arts, social practice, traditional handicrafts, traditional medicine, folk culture, etc.
Distribution	The distribution characteristics and area of Chongqing ICH are analyzed, providing an important reference for subsequent protection and utilization.
Value and Protection	The historical, cultural, social, and economic value of Chongqing ICH is evaluated and analyzed, and countermeasures and suggestions for the protection and inheritance of ICH are proposed.
Inheritance	The current situation and problems of ICH inheritance are analyzed, and countermeasures and suggestions for the protection and inheritance of ICH are put forward.
Utilization	The utilization and development of ICH are proposed, covering ICH tourism development, cultural and creative product development, ICH digital display, etc.

The digital protection and inheritance of ICH is not only an important topic in the academic field, but also has aroused widespread concern in the commercial market. In the digital age, APP interface design has become an important medium to realize the dissemination, popularization, and commercialization of ICH. This section focuses on the application of ICH in the market, emphasizing its APP interface design for in-depth analysis [55].

In the market, increasingly ICH-related APP emerges, providing users rich ICH content and services. For these apps, the interface design quality is often one of the decisive factors in whether users like to use it [56].

Taking the “Intangible Cultural Heritage Life” APP as an example, the interface design of this APP is very simple and beautiful, and the interface color is mainly warm, giving people a warm and comfortable feeling. The front page has a waterfall layout that displays recommended ICH products and activities so users can quickly find what interests them. The APP combines text, pictures, videos, and other forms on the product and event details page to show the essence and features of ICH products and events [57]. In addition, the APP also offers very practical ICH knowledge learning and examination functions, so that users can learn relevant knowledge in a relaxed and happy atmosphere [58].

(3) Proposal of APP interface design scheme for ICH based on DL

In the process of digital protection and inheritance of ICH, APP interface design has become a crucial link. The wide application of DL technology provides new opportunities and possibilities for the digital protection of ICH. This section focusses on the design scheme of APP interface based on DL, aiming at providing an innovative method to better present, spread and protect ICH.

**Fig. 4:**
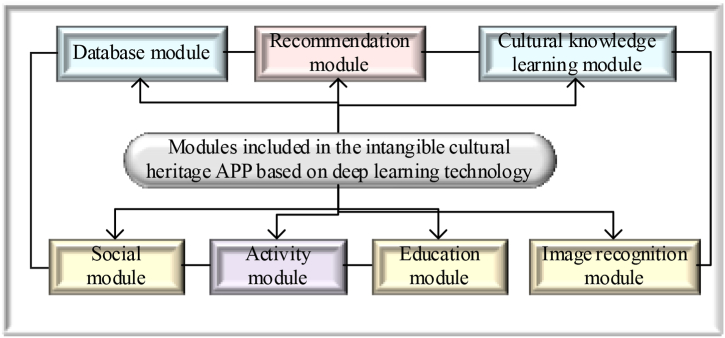
Modules included in the APP of ICH using DL technology.

Given the above research, the designed ICH-related APP based on DL technology contains modules as drawn in Fig. 4.

Fig. 4 shows how the core module may provide users with a complete and multi-dimensional ICH learning and experience platform by combining acceptable interface design and interaction methods with the picture recognition function of DL technology, while encouraging ICH protection and inheritance.

**Fig. 5:**
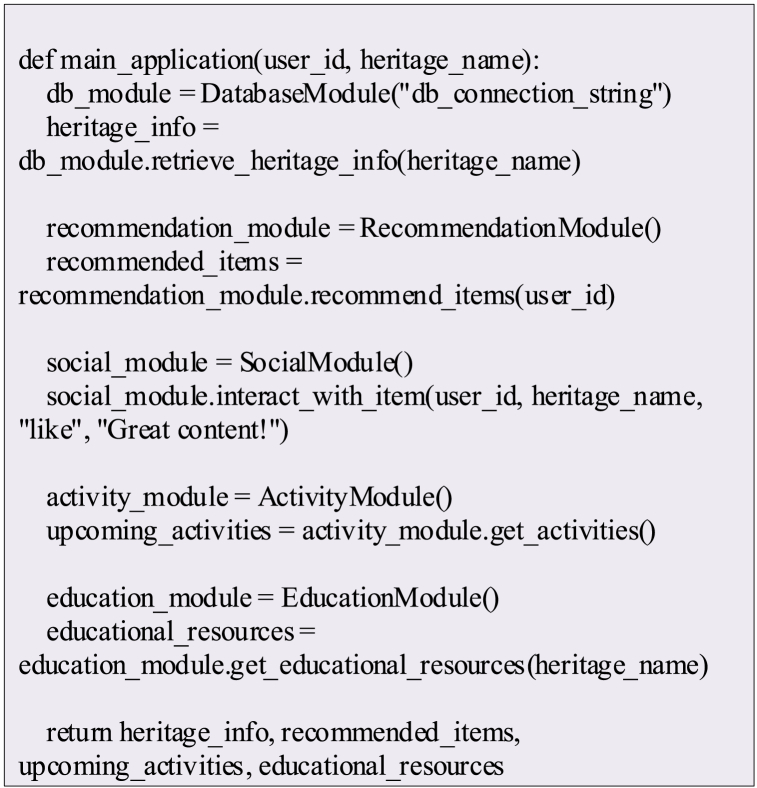
Pseudo-code of the module.

The modules contained in the above-mentioned ICH-related applications designed based on digital library technology are all implemented by Python software, and their pseudo-code is shown in Fig. 5.

In Fig. 5, the Database Module is responsible for connecting to the database and retrieving cultural heritage information. The Recommendation Module is responsible for initializing the recommendation engine and suggesting items for users. Social Module allows users to like and comment on projects. Activity Module is responsible for retrieving upcoming activities. Education Module is responsible for obtaining educational resources related to cultural heritage. The main application function demonstrates how to integrate these modules for users, retrieve cultural heritage information, provide recommendations, enable social interaction, and provide information about upcoming activities and educational resources.

### 3.3 The implementation and application of the chongqing ICH APP interface system

As a region with rich ICH, Chongqing has been actively exploring how to protect, inherit and spread these valuable cultural elements. Therefore, this section adopts modern technology to realize the digital protection and inheritance of ICH, and develops a special APP interface system [59].

According to the above scheme design content, the Chongqing ICH APP interface system uses DL technology, user habits, and visual feelings. The system architecture and design scheme are shown in Table 6.

**Table 6:** Architecture and design scheme of the Chongqing ICH APP interface system.

Module name	Description	Technical tools	Implementation plan
Frontend interface	It displays the APP's main functional modules and interaction design, including the home page, classified browsing, search, collection, sharing, etc.	JavaScript	Modern front-end frameworks are used to improve development efficiency; The user experience is optimized through A/B testing.
Backend data	It includes related ICH data and user data.	Structured Query Language (MySQL)	The appropriate database type, technical storage, and query data are selected.
Search engines	It implements the search function in the APP, searching for relevant ICH content through keywords.	Elasticsearch	Suitable indexes and tokenizers are built to improve search accuracy.
Recommendation engine	Based on user behavior and interests, personalized recommendations are realized, and ICH content aligns with their needs and interests.	Python	The DL framework is chosen to train and optimize the model.
DL model	It realizes the recognition and classification of ICH images and audio.	TensorFlow	The proper DL framework and model are selected to train and optimize the model.
Database Management	It implements data storage and management, including ICH-related data and user data.	MySQL	Database parameters and buffering mechanisms are configured to improve database performance and reliability.

## 4 Experimental design and performance evaluation

This section verifies the performance and feasibility of the ICH image recognition algorithm based on CNN proposed in the previous chapter through experiments, including objective experiments and subjective experiments. The design and implementation of this experiment not only helps to verify the actual effect of our proposed CNN algorithm, but also provides a deep understanding of the application potential of this algorithm in the protection of ICH. Through objective experiments, people can measure the performance of the algorithm in accuracy, recall, F1 value and other performance indicators, thus confirming its recognition ability. Through subjective experiments, people can understand how users perceive and accept this technology to better meet their needs. In addition, the results of these experiments directly affect the further improvement and application of the interactive design of the non-legacy APP interface, which helps promote the development of the field of ICH protection.

### 4.1 Datasets collection

To verify the feasibility of the Chongqing ICH APP interface system scheme and its effect, the dataset and evaluation methods used in this study are as follows:

(1) To explore the effect of the DL-based ICH image recognition model, this study uses the Chinese Cultural Heritage Image (CHIN–CHI) dataset. This dataset was created by the Department of Computer Science and Engineering of a Chinese University in collaboration with the Palace Museum, which contains about 50,000 rich images of Chinese cultural heritage, covering various fields such as painting, sculpture, and handicrafts. These images represent multiple aspects of traditional Chinese culture, including calligraphy, paintings, utensils, etc.

In addition, this study selects 1000 ICH images of 10 different types from this dataset for experiments, of which the number of verification sets, training sets, and test sets accounts for 15 %, 70 %, and 15 %.

(2) A 5-point subjective evaluation questionnaire was used to test the subjective evaluation results of the Chongqing ICH APP interface system. The experiment adopts a randomized group design, and the experimental subjects are randomly divided into an experimental group and a control group to ensure the credibility and reliability of the experimental results. The experimental process includes using the APP for operations, filling out questionnaires, and other steps. The time to use the APP is 30 min, and the questionnaire is filled out after the operation to obtain the user's subjective evaluation of the APP interface design.

### 4.2 Experimental environment

(1) Performance verification of ICH image recognition model

In the process of digital protection and inheritance of ICH, the performance verification of image recognition model is very important. This section focuses on verifying the performance of ICH image recognition model based on DL technology to ensure its effectiveness and reliability in practical application.

In the experiment of this study, a computer equipped with an Intel Core i7 8-core processor and 16 GB RAM was used as the experimental hardware environment. In addition, the NVIDIA GeForce RTX 2080 graphics processor (GPU) was used to accelerate the training and inference process of the DL model, and 1 TB SSD storage was equipped to store experimental data and models. The Windows 10 operating system was used in terms of software environment, and Python 3.8 was configured as the main programming language. In the aspect of the DL framework, TensorFlow 2.5.0 and PyTorch 1.9.0 were employed to construct and train the model, while Scikit-learn 0.24.2 was also used for data analysis. Jupyter Notebook 6.3.0 was utilized to write and run experimental code, while CUDA Toolkit 11.1 was applied for GPU-accelerated computing. Regarding image processing, OpenCV was used for image preprocessing and processing operations.

(2) The control experiments

This section discusses the design and execution of control experiments to ensure that the performance of the model can be accurately evaluated and the results under different experimental conditions are compared.

In the control experiment, the hardware and software environment configuration information used here is presented in Table 7.

**Table 7:** Hardware and software configuration information of the control experiment.

Parameter name	Parameter setting
Smartphone resolution	1080 × 2160
Eye tracker frequency	1000 Hz
Experimental duration	30 min
Front-end development language	JavaScript
Backend development language	MySQL

### 4.3 Parameters setting

According to the previous successful case study of image recognition algorithm based on CNN and the actual demand of this study, the final selection results of specific parameters of CNN constructed in this experiment are shown in Table 8.

**Table 8:** Hyperparameter setting of CNN network.

Layer	Layer type	Convolutional kernel size	Number of convolutional kernels	Step size	Activation function	Pooled Size
1	Convolutional layer	3 × 3	32	1	ReLU	2 × 2
2	Convolutional layer	3 × 3	64	1	ReLU	2 × 2
3	Convolutional layer	3 × 3	128	1	ReLU	2 × 2
4	FC layer	–	256	–	ReLU	–
5	FC layer	–	128	–	ReLU	–
6	Output layer	–	Number of categories	–	Softmax	–

### 4.4 Performance evaluation

(1) System performance verification
1) ICH image recognition effect based on CNN

**Fig. 6:**
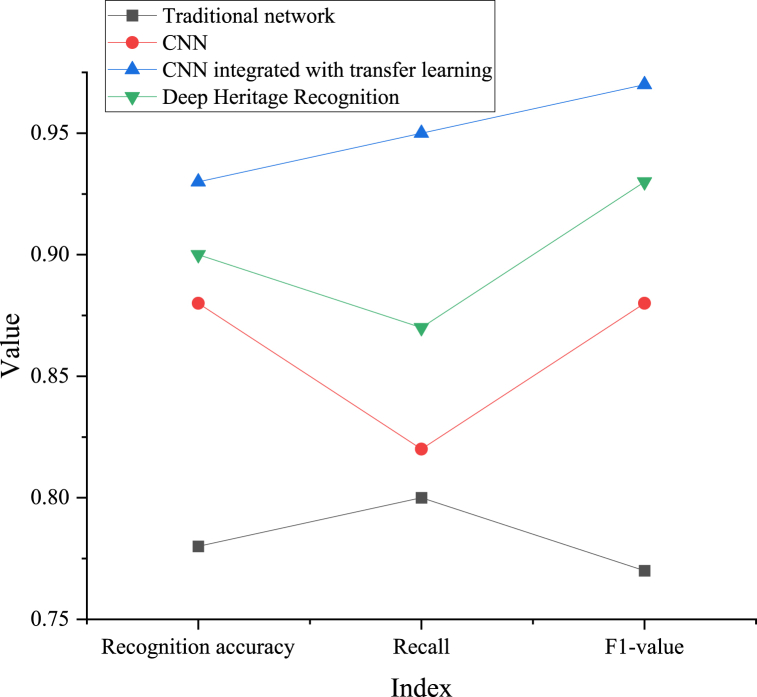
Image recognition effect of ICH using DL.

To evaluate the performance of the Chongqing ICH APP interface system, this study explores the recognition effect of the image recognition module in the system, that is, the performance of the CNN-based ICH image recognition model in recognition accuracy, recall, and F1 value. Among them, the accuracy refers to the ratio of the number of positive and negative samples correctly predicted by the classifier to the total number of samples. The recall rate represents the ratio of the number of positive cases successfully predicted by the classifier to the total number of actual positive cases, which is also called the true case rate. F1 value is a measure that comprehensively considers the accuracy and recall, and is used to balance the performance of the classifier, especially for imbalanced datasets. The model comparison method is used to study the effect of the image recognition model of ICH based on DL, and the results are displayed in Fig. 6. Among them, the traditional recognition network method represents the template matching method, which is a method for comparing the similarity between the template (or sample image) and the input image.

Fig. 6 shows that the ICH image recognition model using CNN performs better than the traditional recognition model and the new Deep Heritage Recognition model in recognition accuracy, recall and F1 value. In addition, by integrating TL into CNN model, the recognition accuracy, recall rate and F1 value of the model are further improved. This shows that CNN combined with TL has a good effect on ICH image recognition task, which is helpful to improve the performance of the model and better recognize ICH images.

2) K-fold cross-validation experimental results

In order to better verify the performance of the model, this paper also uses the K-fold cross-validation method to evaluate the performance of the CNN model. This method is a standard practice to improve the accuracy and robustness of the model. Firstly, this paper divides the dataset into five subsets and conducts many experiments. In each experiment, one subset is used for verification and the rest is used for training. Secondly, this paper repeats this process five times, each time using a different subset as the verification set. Finally, the results of these five experiments are averaged to get the final performance evaluation results as shown in Table 9.

**Table 9:** Experimental results of K-fold cross-validation.

Index	Subset 1	Subset 2	Subset 3	Subset 4	Subset 5	Average value
Accuracy (%)	95.2	94.5	94.8	95.0	94.7	94.84
Recall rate (%)	94.7	94.3	94.6	94.8	94.5	94.58
F1 value (%)	94.9	94.4	94.7	94.9	94.6	94.70

Table 9 shows that the ICH image recognition model proposed in this paper has achieved relatively consistent performance evaluation on five different data subsets. The accuracy of these subsets is stable, with an average of 94.84 %, which shows that the model has high accuracy in identifying ICH images. The recall rate and F1 value also show similar stability, with an average of 94.58 % and 94.70 % respectively. The above results emphasize the consistency of the performance of the model on different data subsets, and its effectiveness and robustness in the image recognition task of ICH.

(2) Subjective evaluation of the Chongqing ICH APP interface system
1) The results of the principal component analysis (PCA) of the questionnaire

**Fig. 7:**
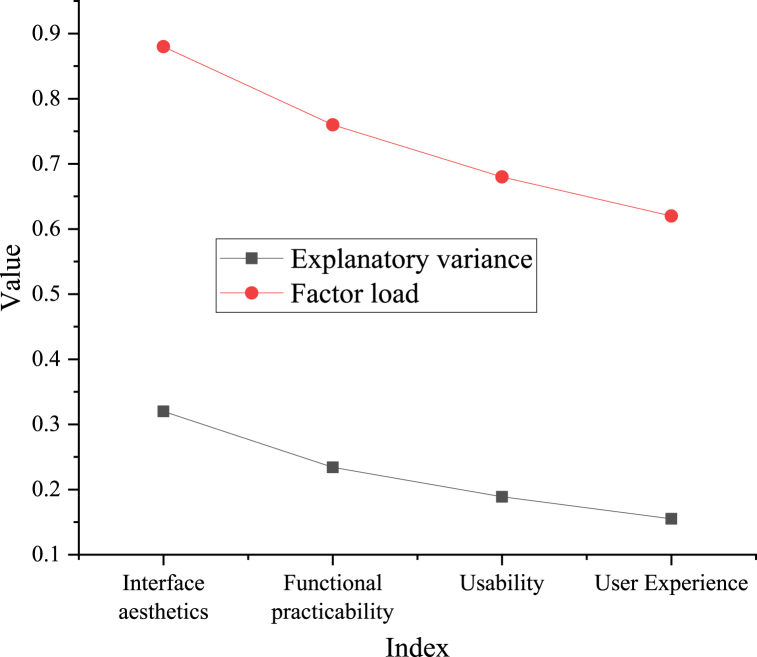
PCA results of the questionnaire.

The results obtained after analyzing the questionnaire data based on the PCA are portrayed in Fig. 7.

Fig. 7 shows that in the questionnaire results, consumers' attention to APP interface design is mostly spread in four areas: interface aesthetics, functional practicability, usability, and user experience. The most significant influence is interface aesthetics, which accounts for 32.1 % of total variance.

2) Comparative experimental results

**Fig. 8:**
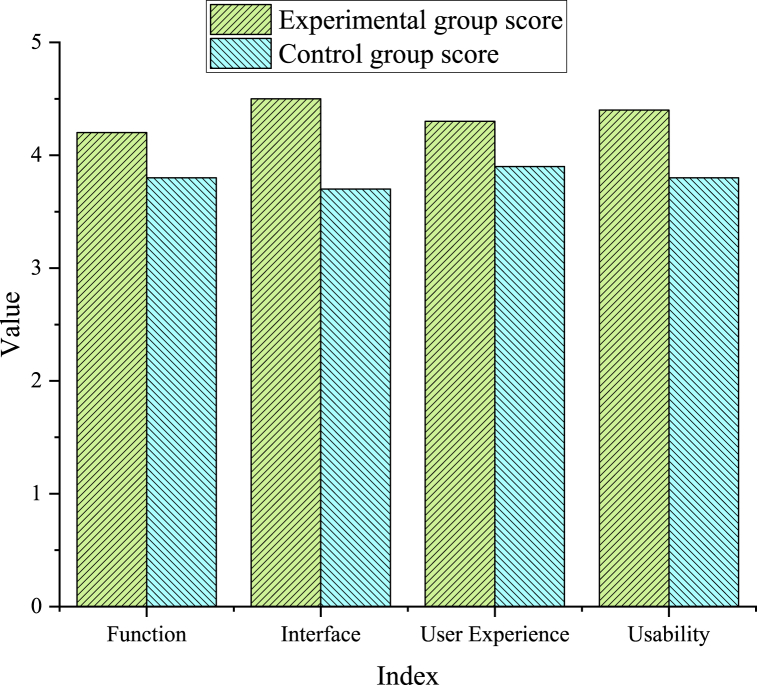
User experience scores of each module of the interface system.

The user experience score of the designed interface system is obtained using the experimental control method, as demonstrated in Fig. 8.

Fig. 8 denotes that the experimental group scored higher than the control group in function, interface, user experience, and usability. This means that based on DL technology, user habits, and visual feelings, the Chongqing ICH APP interface system performs better in user experience and usability.

### 4.5 Discussion

Here, the ICH image recognition model based on CNN and the Chongqing ICH APP interface system are studied. Compared with previous studies, this study has made breakthroughs in the following aspects. In the aspect of ICH image recognition, an experimentation-based comparison demonstrates that the model utilizing CNN outperforms the model without CNN in recognition accuracy, recall, and F1 value. Furthermore, the TL-based model exhibits further enhancements across these indicators. This indicates that CNN and TL have a good effect on the ICH image recognition task and can improve model performance. In the aspect of the designed APP interface system, the experimental results present that the interface system based on user habits, DL technology, and visual feelings evidences superior performance in usability, user experience, and other aspects. According to PCA's analysis of questionnaire data, consumers' attention to the APP interface design is primarily focused on four aspects: usability, interface aesthetics, user experience, and functional practicality, with interface aesthetics emerging as the most influential determinant. In conclusion, a series of effective methods and technologies are adopted in this study to explore and optimize ICH image recognition and APP interface design. The experimental results testify that these techniques and methods can enhance the model's and system's performance, thus improving user experience and satisfaction. Future studies can further explore and optimize these methods to make them more applicable to a broader range of application scenarios and promote the conservation and inheritance of ICH.

To sum up, the discussion on the image recognition model of ICH based on CNN and Chongqing ICH APP interface system shows a wide range of practical value and applicability. This study provides new methods and tools for the protection and inheritance of cultural heritage, and provides strong support in the field of digital cultural heritage protection and inheritance. However, there are still some problems in management and decision-making in the field of ICH protection and digital inheritance. Therefore, this study provides some suggestions. Firstly, managers should realize the potential of modern technology, especially DL technology, in the protection and inheritance of ICH, and need to actively explore ways to combine technology with cultural inheritance to better spread and protect traditional culture. Secondly, when creating ICH-related apps and interfaces, user experience should be given top consideration. By making sure the interface is simple to use, appealing, and meets user expectations, more users will be encouraged to engage with cultural heritage. Thirdly, TL is an effective method to improve the performance of the model. People can consider introducing TL into the project to speed up model training and improve the effect of image recognition. Community participation and cooperation is also the key to the successful protection and inheritance of ICH. Managers should encourage the community to actively participate and ensure that the protection work is the common cause of the whole society. It is also the key to improve the protection effect of cultural heritage by constantly improving technology and methods, comparing, and studying different models and methods, and constantly optimizing and adapting to different application scenarios. Finally, sustainability and environmental factors should be considered in ICH protection and inheritance projects to ensure that the protection work is coordinated with social and economic development and does not have a negative impact on the environment. In addition, in the ICH protection and digital heritage project, it is important to establish an effective monitoring and evaluation mechanism to track the progress and effect of the project. This can be achieved through regular evaluation and feedback to ensure that the project achieves the expected goals. Managers need to continue to pay attention to the operation of the project and find and solve potential problems in time. In addition, financial and resource management are also key issues. Managers need to allocate budgets and resources effectively to ensure the sustainability and long-term success of the project. This requires comprehensive consideration of all aspects of the project, including technical input, human resources, equipment and maintenance costs, to ensure that the project is not plagued by resource shortage. Finally, the protection of cultural heritage needs the cooperation of government, community and academia. Managers should actively promote cross-departmental cooperation and encourage information sharing and resource integration among different stakeholders. Only through joint efforts can the protection and inheritance of cultural heritage be better realized, and the goals of sustainability and social sharing can be achieved.

## 5 Conclusion

This study proposes an image recognition model of ICH based on TL and CNN, and applies it to the image recognition task of ICH in Chongqing. The experimental results show that compared with other traditional algorithms and new algorithms, the algorithms based on TL and CNN have higher recognition recall, accuracy and F1 value (0.927, 0.948 and 0.96, respectively). Meanwhile, the Chongqing ICH APP interface system based on visual perception, DL technology and user habits is designed and evaluated. The evaluation results show that the experimental group scores higher than the control group in terms of function, interface, usability and user experience, which not only emphasizes the feasibility of our interface system, but also highlights its importance in providing user-friendly experience. To sum up, this study has great influence and potential value in the field of ICH protection and inheritance. By putting forward the image recognition model of ICH based on TL and CNN and the innovative Chongqing ICH APP interface system, new vitality is injected into the digital protection and inheritance of cultural heritage, and further promoted the sustainable development and inheritance of ICH. This study provides a solid foundation for further exploration in related fields in the future.

Although this study has made remarkable progress in the field of ICH protection and inheritance, there are still some potential limitations. On the one hand, although the model in this study shows good performance, there is still room for further optimization, including trying more different DL models and TL strategies to improve the robustness of performance. On the other hand, although the user experience and the aesthetic feeling of the interface have been concerned, more in-depth research is still needed to meet the needs of the majority of users. Therefore, in the future, this study will further explore the comparative study of different CNN architectures and other DL models to find the most suitable model for different application scenarios. In addition, this study will further optimize the TL method and improve the knowledge transfer strategy to improve the generalization performance of the model. Meanwhile, the user feedback and interface aesthetics are deeply studied to further improve the user experience to improve the effect of cultural heritage protection and inheritance.

## Funding

This research was supported by the research project of Chongqing Technology and Business University as below:1. "Research on the Regeneration of Chongqing Traditional Culture in Environmental Design" (Grant No: 2055018) .2.“Research on the Revitalization of Third Line Architectural Heritage Landscape in Southwest China from the Perspective of Cultural Revitalization Theory”(Grant No. 2151024).

## Data availability statement

Data will be made available on request.

## CRediT authorship contribution statement

**Yanlong Liu:** Writing – original draft, Methodology, Formal analysis, Data curation, Conceptualization. **Peiyun Cheng:** Writing – review & editing, Validation, Supervision, Software, Investigation. **Jie Li:** Visualization, Validation, Formal analysis.

## Declaration of competing interest

The authors declare the following financial interests/personal relationships which may be considered as potential competing interests:

## Appendix

**Questionnaire: Evaluation of the APP Interface Design Scheme for Intangible Cultural Heritage in Chongqing**.

To improve and optimize the APP interface design scheme of the intangible cultural heritage in Chongqing, we invite you to participate in this questionnaire survey. Please answer the following questions truthfully based on your experience and feelings. Your opinions and suggestions are crucial for improving the quality of the APP interface design. Thank you for your participation!

### I Personal information (optional)

1. Gender:
  -Male
  - Female
  - Other
2. Age:
  - Under 18 years old
  − 18-25
  − 26-35
  − 36-45
  - Over 46
3. Occupation: ____________

### II Experimental process experience

4. Have you ever used a similar intangible cultural heritage APP?
  - Yes
  - No
5. The APP you used in this experiment is:
  - Chongqing intangible cultural heritage APP interface system based on deep learning technology and user habits and visual perception
  - Other similar apps in the market (such as “China Intangible Cultural Heritage")
6. What improvements or innovations have you experienced during the operation of the APP? (Open questions)

### III Subjective evaluation

Please rate the following items on a scale of 1 (very dissatisfied) to 5 (very satisfied).

7. Functional integrity:
  − 1
  − 2
  − 3
  − 4
  − 5
8. Interface aesthetics:
  − 1
  − 2
  − 3
  − 4
  − 5
9. User experience:
  − 1
  − 2
  − 3
  − 4
  − 5
10. Usability:
  − 1
  − 2
  − 3
  − 4
  − 5

### IV Opinions and suggestions

11. What aspects do you think can be further improved in the Chongqing Intangible Cultural Heritage APP interface system? (Open questions)
12. Are you willing to continue using and recommending this app to others?
  - Yes
  - No

Thank you for your participation and valuable feedback! Your feedback will contribute to further enhancing the design and functionality of the Chongqing Intangible Cultural Heritage App interface system.
